# Efficient Conversion of Astrocytes to Functional Midbrain Dopaminergic Neurons Using a Single Polycistronic Vector

**DOI:** 10.1371/journal.pone.0028719

**Published:** 2011-12-09

**Authors:** Russell C. Addis, Fu-Chun Hsu, Rebecca L. Wright, Marc A. Dichter, Douglas A. Coulter, John D. Gearhart

**Affiliations:** 1 Institute for Regenerative Medicine and Department of Cell and Developmental Biology, University of Pennsylvania School of Medicine, Philadelphia, Pennsylvania, United States of America; 2 Mahoney Institute of Neurological Sciences, University of Pennsylvania School of Medicine, Philadelphia, Pennsylvania, United States of America; 3 Department of Neurology, University of Pennsylvania School of Medicine, Philadelphia, Pennsylvania, United States of America; 4 Departments of Pediatrics and Neuroscience, University of Pennsylvania School of Medicine, Philadelphia, Pennsylvania, United States of America; 5 Division of Neurology, Children's Hospital of Philadelphia, Philadelphia, Pennsylvania, United States of America; University of California San Diego, United States of America

## Abstract

Direct cellular reprogramming is a powerful new tool for regenerative medicine. In efforts to understand and treat Parkinson's Disease (PD), which is marked by the degeneration of dopaminergic neurons in the midbrain, direct reprogramming provides a valuable new source of these cells. Astrocytes, the most plentiful cells in the central nervous system, are an ideal starting population for the direct generation of dopaminergic neurons. In addition to their potential utility in cell replacement therapies for PD or in modeling the disease *in vitro*, astrocyte-derived dopaminergic neurons offer the prospect of direct *in vivo* reprogramming within the brain. As a first step toward this goal, we report the reprogramming of astrocytes to dopaminergic neurons using three transcription factors – ASCL1, LMX1B, and NURR1 – delivered in a single polycistronic lentiviral vector. The process is efficient, with 18.2±1.5% of cells expressing markers of dopaminergic neurons after two weeks. The neurons exhibit expression profiles and electrophysiological characteristics consistent with midbrain dopaminergic neurons, notably including spontaneous pacemaking activity, stimulated release of dopamine, and calcium oscillations. The present study is the first demonstration that a single vector can mediate reprogramming to dopaminergic neurons, and indicates that astrocytes are an ideal starting population for the direct generation of dopaminergic neurons.

## Introduction

Parkinson's Disease (PD) is marked by progressive loss of dopaminergic neurons in the ventral midbrain. Although the somata of these neurons are located in the substantia nigra, it is their projections to the striatum that release dopamine to mediate motor control. For this reason, the caudate and putamen regions of the striatum have been the primary targets for cell replacement strategies in PD [1]. Restoring dopaminergic tone to the striatum via the engraftment of dopaminergic neurons has long been a goal in the field of regenerative medicine, beginning with the transplantation of fetal mesencephalic tissue [2]–[6]. Given the conflicting results of these studies, as well as the difficulty in obtaining sufficient quantities of fetal tissue, alternative cell sources have been pursued (well-reviewed in [7]). Both neural stem cells [8] and embryonic stem cells [9] have shown great promise in their ability to differentiate into dopaminergic neurons, while the advent of induced pluripotent stem (iPS) cells made real the possibility of generating patient-specific stem cell lines [10]. More recently, direct reprogramming has demonstrated that stem cells may not be necessary at all – three groups have reported that ectopic expression of small sets of transcription factors can directly convert fibroblasts to dopaminergic neurons [11]–[13]. Astrocytes are an attractive alternative to fibroblasts as a starting population of cells for reprogramming to dopaminergic neurons. Previous studies have demonstrated that astrocytes can be directly reprogrammed to neurons that form functional synapses [14], [15]. Conversion of astrocytes to dopaminergic neurons would not only provide a new source of neurons for use in cell-based therapies for PD, but this approach also raises the possibility of direct *in vivo* reprogramming as a novel treatment strategy [16]. Since this virus-based strategy would require no cellular transplantation, many of the concerns of immunological rejection in cell replacement therapies would be negated. Furthermore, there is now considerable evidence that reprogrammed cells retain an epigenetic memory of their original cell type [17]–[19], and iPS cells derived from astrocytes have a greater propensity for neuronal differentiation than those derived from fibroblasts [20]. Thus, the developmentally close relationship of astrocytes to neurons may prove advantageous to effective reprogramming. In the present study, we report the direct conversion of astrocytes to dopaminergic neurons via three transcription factors, with the development of a polycistronic lentiviral vector to facilitate future efforts at *in vivo* reprogramming.

## Results

### Transcription factor screen and polycistronic vector generation

**Figure 1:**
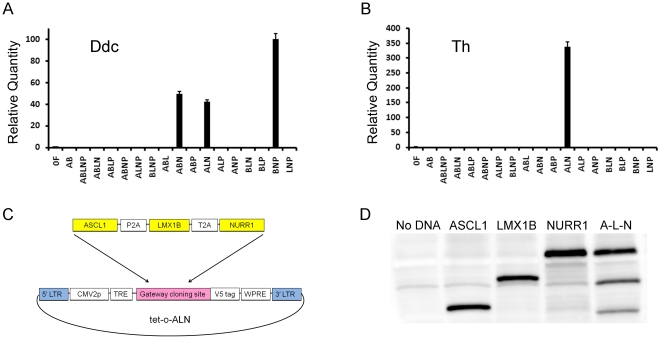
Transcription factor screen and polycistronic vector construction. (A–B) Evaluation of transcription factor combinations to induce reprogramming. RT-PCR results for a subset of the 74 transcription factor combinations tested for their ability to induce expression of DOPA decarboxylase (Ddc) and tyrosine hydroxylase (Th) in mouse embryonic fibroblasts after 7 days of factor expression. 0F: uninfected control; A: ASCL1; B: BRN2; L: LMX1B; N: NURR1; P: PITX3. The combination of ASCL1, LMX1B, and NURR1 (ALN) was the only combination to give robust expression of both Ddc and Th. (C) Polycistronic vector containing open reading frames of human ASCL1, LMX1B, and NURR1 linked by viral 2A sequences. (D) Complete cleavage at 2A peptide sites confirmed by *in vitro* transcription and translation (TnT) of lentiviral plasmids in the presence of biotinylated lysine. Streptavidin-HRP Western blot shows all newly synthesized protein. Lane 1: No DNA TnT control. Lanes 2–4: TnT performed on original single-factor plasmids. Lane 5: TnT for polycistronic ALN plasmid. Band intensity is proportional to the number of lysine residues present in each protein sequence.

To identify a combination of transcription factors that is sufficient to mediate reprogramming to dopaminergic neurons, twelve transcription factors known to play critical roles in midbrain dopaminergic neuron development and/or maintenance [21]–[23] were cloned into the doxycycline-inducible lentiviral vector FU-tetO-Gateway (Figure 1A). Seventy-four unique combinations of these vectors were used to transduce mouse embryonic fibroblasts for the initial factor screen. RNA of transduced cells was harvested after 7 days of doxycycline-induced factor expression and assayed via RT-PCR for expression of tyrosine hydroxylase (Th) and DOPA decarboxylase (Ddc), the enzymes of dopamine synthesis. All experiments were performed in triplicate. The three-factor combination of ASCL1, LMX1B, and NURR1 resulted in the most robust expression of Th and Ddc (Figure 1A–B). This combination was selected for further analysis.

In order to increase the efficiency of cells receiving all three transcription factors, as well as to reduce variability resulting from different ratios of the factors reaching individual cells, we constructed a polycistronic vector. The open reading frames of ASCL1, LMX1B, and NURR1 were linked via recombinant PCR such that viral 2A peptide sequences [24] separate the three genes, as shown in Figure 1C. A glycine-serine-glycine (GSG) linker was included upstream of each 2A sequence to facilitate protein cleavage. The ASCL1-P2A-LMX1B-T2A-NURR1 cassette (hereafter referred to as ALN) was inserted into FU-tetO-Gateway to produce the tetO-ALN vector. Cleavage at the 2A sites was validated by performing *in vitro* transcription and translation using the T7 promoter located at the start of the Gateway recombination site in the presence of biotinylated lysine. A streptavidin-HRP western blot confirmed complete cleavage at both the T2A and P2A sites (Figure 1D). The tetO-ALN lentiviral vector delivered ALN to both astrocytes and fibroblasts at >99% efficiency, as determined by immunocytochemistry for the C-terminal V5 tag on NURR1 (data not shown).

### Characterization of gene and protein expression in induced dopaminergic neurons

**Figure 2:**
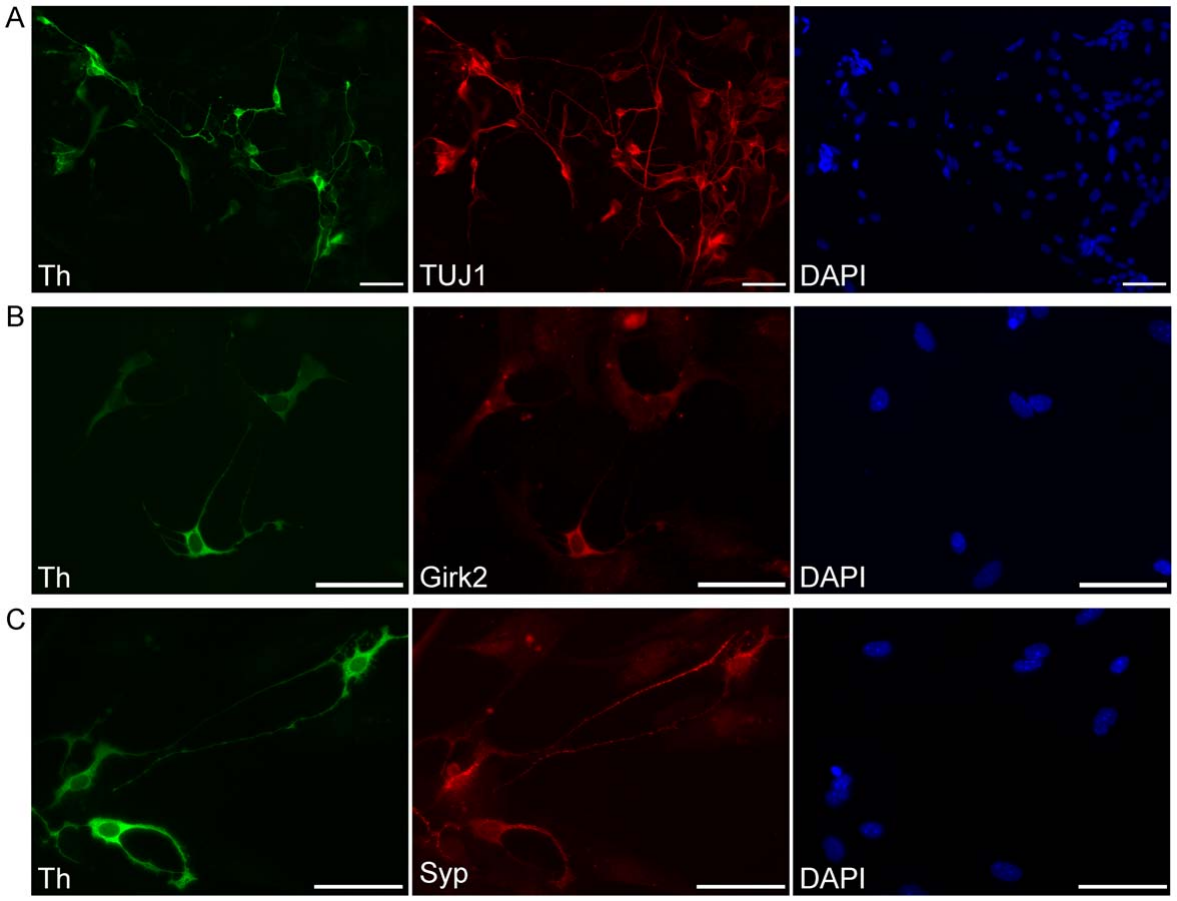
Immunocytochemical characterization of astrocyte-derived dopaminergic neurons. Astrocyte-derived dopaminergic neurons, 17 (A) to 19 (B,C) days post-induction. (A) Efficient conversion of astrocytes to dopaminergic neurons demonstrated by immunocytochemistry for the neuronal marker TUJ1 (type III beta tubulin) and the dopaminergic marker tyrosine hydroxylase (Th); the pan-nuclear marker DAPI is also shown. (B) Th positive neurons express the potassium channel Girk2, a marker of A9 (substantia nigra) dopaminergic neurons. (C) Punctate synaptophysin (Syp) expression in tyrosine hydroxylase (Th) positive neurons, indicating the potential to form synaptic connections. Scale bars 50 µm.

Astrocytes were transduced with FUdeltaGW-rtTA (the reverse tetracycline transactivator, [25]) and tetO-ALN. The following day, doxycycline was added to astrocyte medium to induce ALN expression (Day 0). After four days in astrocyte medium with doxycycline, transduced cells were switched to NB27G neuronal medium. Doxycycline was removed on Day 10. After 14 days from the initial ALN induction, 35.1±1.5% of cells expressed type III beta-tubulin (clone TUJ1), a neuronal marker. 50.9±3.3% of TUJ1+ cells were also positive for tyrosine hydroxylase, yielding an overall conversion rate of 18.2±1.5% (Figure 2A). Quantification was performed by counting a total of 3357 cells in three independent reprogramming experiments.

**Figure 3:**
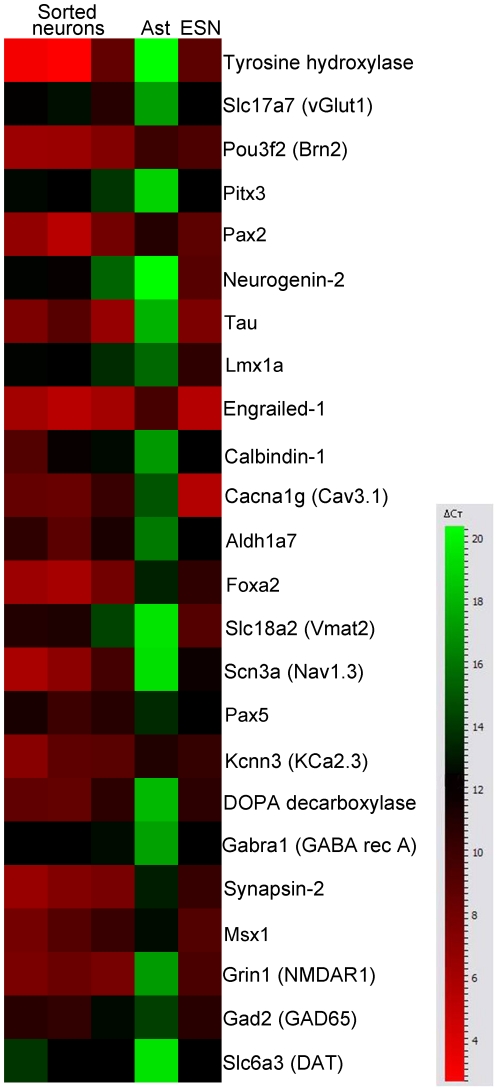
Transcriptional profile of astrocyte-derived dopaminergic neurons. Heat map of quantitative RT-PCR results comparing astrocyte-derived neurons magnetically sorted for a *MAP2*-CD4 reporter. Ast, uninfected astrocytes. ESN, mouse embryonic stem cell-derived neurons, generated via co-culture with PA6 stromal cells. Induced dopaminergic neurons express markers consistent with midbrain dopaminergic neurons. Color scale indicates change in Ct (threshold cycle) relative to the normalizing actin control. Higher delta Ct values correspond to lower relative gene expression, with every Ct decrease of 3.3 representing a ten-fold increase in relative expression.

We constructed a lentiviral reporter vector in which the cell surface marker, CD4, is expressed under the control of the neuron-specific *MAP2* gene promoter. To further characterize the induced dopaminergic neurons, we sorted ALN-derived neurons on Day 14 using the *MAP2*-CD4 reporter vector and magnetic beads conjugated to anti-CD4. RNA was isolated from sorted cells and assayed via RT-PCR for expression of a panel genes shown in Figure 3. Gene expression in sorted neurons was compared to astrocytes that did not receive ALN as well as mouse embryonic stem cells that had been differentiated to dopaminergic neurons via co-culture with PA6 stromal cells [26]. Sorted neurons displayed robust upregulation of genes expressed in midbrain dopaminergic neurons such as Pitx3, Lmx1a, Engrailed-1, aldehyde dehydrogenase, Foxa2, the vesicular monoamine transporter Vmat2, Msx1, and the dopamine transporter. Immunocytochemistry revealed expression of Girk2, a potassium channel widely expressed in A9/substantia nigra dopaminergic neurons [27], in virtually all (>99%) of the Th-immunoreactive cells (Figure 2B). Otx2, a protein expressed in mature A10/ventral tegmental area dopaminergic neurons [28], [29], was not detected. Induced dopaminergic neurons exhibited robust synaptophysin expression (>99% of Th-immunoreactive cells, Figure 2C), suggesting the capacity to form synaptic connections.

### Functional characterization of induced dopaminergic neurons

**Figure 4:**
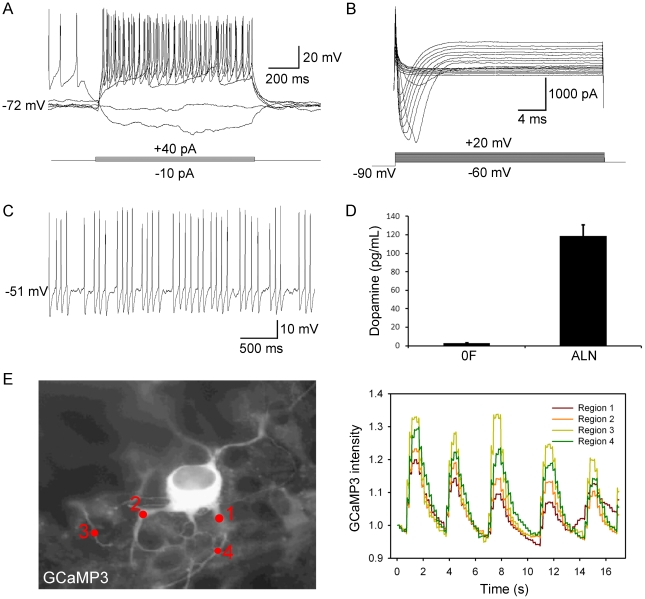
Functional characterization of astrocyte-derived dopaminergic neurons. (A) Action potential firing characteristics of induced dopaminergic neurons. Five overlapping traces are depicted derived from whole-cell current clamp recording of a representative induced dopaminergic neuron, elicited in response to hyperpolarizing and depolarizing current injection, increased from −10 to +40 pA in 10 pA increments. (B) Voltage-dependent sodium currents in induced dopaminergic neurons. Membrane potential was initially held at −90 mV and incrementally increased from −60 to +20 mV in 5 mV depolarizing steps. (C) Spontaneous action potential firing, consistent with a dopaminergic neuron pacemaker phenotype. The recording was conducted at resting membrane potential (−51 mV). (D) Dopamine release quantified by HPLC. Membrane depolarization was induced with 56 mM KCl at 17 days post-infection. 0F: uninfected astrocytes; ALN: induced dopaminergic neurons. (E) *MAP2*-GCaMP3 reveals rhythmic oscillations of intracellular calcium. Left panel displays a single frame of Movie S1, a recording of oscillating levels of intracellular calcium, which is presented as a histogram of GCaMP3 fluorescence intensity in the adjacent panel.

To evaluate the electrophysiological phenotype of induced dopaminergic neurons, patch clamping was performed on induced neurons between days 9 and 26 of initial ALN induction. Neurons were identified by screening for GFP driven by the *MAP2* promoter. Current clamp recordings in Day 9 cells showed an immature spiking pattern – generally a single action potential per step current injection. By Day 14–21, neurons demonstrated repetitive firing of action potentials in individual step current injections (Figure 4A). Of 30 patched cells, 24 fired action potentials (80%). Recorded cells had an average resting membrane potential of −55.4 mV. In voltage clamp, large sodium and potassium currents were seen (Figure 4B), with an average maximum I_Na_ of 1546 pA. Electrophysiological properties of induced dopaminergic neurons are summarized in Table 1. Recordings were made on neurons generated in four independent reprogramming experiments.

**Table 1:** Electrophysiological properties of astrocyte-derived dopaminergic neurons.

	Capacitance (pF)	RMP (mV)	Spontaneous firing (%)	AP freq (Hz)	Threshold (mV)	I_Na_-max (pA)	I_K_-max (pA)	IR (GΩ)
Mean	16.22	−55.39	43.00	5.60	−27.73	1546.00	1381.91	1.48
SEM	0.76	1.46	11.00	1.17	2.27	175.21	151.51	0.23
N	8	16	21	7	11	11	11	16

RMP: resting membrane potential; AP freq: frequency of spontaneous action potentials; I_Na_ and I_K_: Maximum sodium and potassium currents; IR: input resistance.

It has been well established that midbrain dopaminergic neurons are pacemaker neurons, spontaneously firing action potentials at a rate between 1 and 9 Hz with an average of 4.5±1.7 Hz [30]–[33]. We observed spontaneous firing of action potentials in 43% of recorded *MAP2*-GFP+ induced neurons, a fraction that is consistent with the 50.9±3.3% of neurons that were immunoreactive for tyrosine hydroxylase (Figure 4C). The frequency of spontaneous firing ranged from 0.9 to 8.6 Hz, with an average of 5.6±1.2 Hz. The pacemaker activity of dopaminergic neurons is accompanied by rhythmic fluctuations in intracellular calcium ion concentration [34]–[37]. To determine whether induced dopaminergic neurons exhibit calcium oscillations, we transduced astrocytes (prior to reprogramming) with a lentiviral vector containing the genetically-encoded calcium indicator GCaMP3 [38] driven by the *MAP2* promoter. We detected robust calcium oscillations with an average frequency of 0.49±0.11 Hz (Figure 4E). A typical GCaMP3-expressing induced dopaminergic neuron is shown in Movie S1.

To confirm that induced dopaminergic neurons produce and release dopamine, we stimulated cells with 56 mM KCl and measured dopamine release via HPLC. Cells assayed at 17 days post-transduction with ALN released dopamine in response to membrane depolarization while un-transduced cells (0F) did not, as shown in Figure 4D. No significant levels of epinephrine, norepinephrine, or serotonin were detected.

### Direct reprogramming of fibroblasts to dopaminergic neurons

Given the widespread use of fibroblasts as a starting cell population in reprogramming, we tested whether our polycistronic vector was effective on mouse embryonic fibroblasts. The ALN vector was able to reprogram mouse embryonic fibroblasts to Th+/TUJ1+ neurons, as shown in Figure S1, with an overall efficiency of 9.1±0.9%. Fibroblast-derived dopaminergic neurons exhibited large sodium currents and spontaneous action potential firing (Figure S1B–D). Dopaminergic neurons generated from fibroblasts using a nearly identical set of transcription factors (ASCL1, LMX1A, and NURR1) were recently described and characterized in detail [12].

## Discussion

Our study is the first to demonstrate direct reprogramming of astrocytes to dopaminergic neurons. These neurons exhibit gene and protein expression patterns that are consistent with A9 midbrain dopaminergic neurons. Given the mounting evidence that cells retain some epigenetic memory when driven to pluripotency and subsequent differentiation, such memory is almost certain to be maintained in direct reprogramming. Global epigenetic profiling that compares authentic dopaminergic neurons to those derived from astrocytes and fibroblasts will be informative in determining the relative completeness of these reprogramming processes. It will also be important to compare the ability of astrocyte- and fibroblast-derived dopaminergic neurons to engraft and function in animal models of PD.

In addition to providing a novel source of dopaminergic neurons for use in cell-based therapies for PD, our use of astrocytes as the starting population allows an approach that may obviate the need for grafting altogether – direct *in vivo* reprogramming to replace lost neurons. Such an approach is facilitated by the development of a polycistronic vector, especially since one of the three factors, ASCL1, has been shown to reprogram astrocytes to non-dopaminergic neurons in the absence of additional factors [14]. The fact that the polycistronic ALN vector reprograms astrocytes at such a high efficiency is also noteworthy, given the history of polycistronic vector use in generating iPS cells. A single vector delivering transcription factors that induce pluripotency was shown to be effective, but at a significantly lower rate than that which could be achieved when the factors were delivered individually [39]. This was presumed to indicate that a particular ratio of reprogramming factors is ideal for inducing pluripotency. In the case of reprogramming to dopaminergic neurons, we can conclude that either the ratio of factors is not as important or, less likely, that our polycistronic vector fortuitously delivers the factors in the appropriate proportions. The present study takes a crucial step toward the ultimate goal of *in vivo* reprogramming of astrocytes to dopaminergic neurons. Future work will determine the feasibility of this approach in animal models of PD.

## Materials and Methods

### Lentiviral expression vector construction

To make a drug-inducible lentiviral vector that is compatible with Gateway recombination (Invitrogen), we removed the OCT4 open reading frame from FU-tetO-hOCT4 (Addgene plasmid 19778, [25]) and replaced it with a Gateway cassette cloned from pEF-DEST51 (Invitrogen) to generate FU-tetO-Gateway. ORFs that lack stop codons can be cloned into this plasmid to express proteins with a C-terminal V5 epitope tag. ORFs and cDNAs were purchased from Open Biosystems (Table 2).

**Table 2:** Open Reading Frames and cDNAs cloned into FU-tetO-Gateway.

Transcription Factor	Accession Number	Open Biosystems Clone ID
ASCL1	DQ894571	100009031
BRN2 (POU3F2)	BC051699	4817001
EN1	BC111840	40080843
FOXA2	DQ896132	100010592
LMX1B	EL736297	13957
Msx1	BC016426	4923403
NGN2	BC036847	5247719
NURR1 (NR4A2)	EU832740	100067769
OTX2	EU176492	100011617
PAX2	BC141452	100014752
PAX5	BC156189	100061597
PITX3	DQ895132	100009592

### Reporter vector construction

The *MAP2*-GFP lentiviral reporter plasmid pGZ-hMAP2 was purchased from System Biosciences (SR10047PA-1). The *MAP2* promoter sequence from this plasmid was then cloned along with the Gateway cassette of pEF-DEST51 into the backbone of FUdeltaGW-rtTA (Addgene plasmid 19780) following removal of the ubiquitin promoter-rtTA sequence to produce FU-*MAP2*-Gateway. This destination vector was subsequently used to generate *MAP2*-CD4 (human CD4 sequence cloned from pMACS4-IRESII, Miltenyi Biotec) and to generate *MAP2*-GCaMP3 (GCaMP3 cloned from G-CaMP3, Addgene plasmid 22692). All lentiviruses were packaged in HEK-293T cells using psPAX2 (Addgene plasmid 12260) and pMD2.G (Addgene plasmid 12259). Calcium imaging of neurons containing the *MAP2*-GCaMP3 reporter was performed on an Olympus iX-81 with a 60× oil objective lens using Metamorph and HyperCam software, with neurons grown on poly-D-lysine coated FluoroDishes (World Precision Instruments FD35PDL-100). GCaMP3 movies were analyzed in ImageJ, corrected to account for photobleaching, and intensities were plotted using SigmaPlot.

### Polycistronic vector construction

Open reading frames of human ASCL1, LMX1B, and NURR1 were cloned from the plasmids listed above using the following primers: B1-ASCL1-FWD: GGGGacaagtttgtacaaaaaagcaggctACCATGGAAAGCTCTGCCAAG; ASCL1-REV: CATCTCCTGCTTGCTTTAACAGAGAGAAGTTCGTGGCACCGGATCCGAACCAGTTGGTGAAGTC; LMX1B-FWD: CTCTCTGTTAAAGCAAGCAGGAGATGTTGAAGAAAACCCCGGGCCTATGTTGGACGGCATCAAG; LMX1B-REV: ACGTCACCGCATGTTAGAAGACTTCCTCTGCCCTCTCCGGATCCGGAGGCGAAGTAGGAACT; NURR1-FWD: GAAGTCTTCTAACATGCGGTGACGTGGAGGAGAATCCCGGCCCTATGCCTTGTGTTCAGGCG; B2-NURR1-REV: GGGGaccactttgtacaagaaagctgggtAGAAAGGTAAAGTGTCCAG (extra A added to maintain reading frame with C-terminal V5-tag). The 3 ORFs were then joined via recombinant PCR using B1-ASCL1-FWD and B2-NURR1-REV, cloned into pDONR221, and subsequently cloned into FU-tetO-Gateway via Gateway recombination. Cleavage at 2A sites was verified by performing *in vitro* transcription and translation using the TnT T7 Coupled Reticulocyte Lysate System (Promega L4610) in the presence of biotinylated lysine (Promega L5061). 5 µL of each TnT-generated protein sample was resolved via PAGE, transferred to PVDF membrane, incubated with streptavidin-conjugated horseradish peroxidase (Cell Signaling Technology 3999, 1∶2000), and detected with ECL Plus (GE Healthcare RPN2132).

### Cell culture

Primary mouse embryonic fibroblasts (strain CF-1) were purchased from Millipore (PMEF-CFL) and cultured in DMEM (Gibco) containing 10% fetal bovine serum (Hyclone) and 1× non-essential amino acids (Gibco). Primary postnatal mouse astrocytes (strain CD1) isolated from cerebral cortex were purchased from ScienCell (MA1800) and cultured in Astrocyte Growth Medium (AGM, Lonza). For reprogramming, astrocytes or fibroblasts were plated onto poly-L-lysine-coated glass coverslips or 6-well plates (BD Biocoat). Expression of transcription factors was induced by the addition of doxycycline (2 µg/mL). On Day 4, medium was switched to NB27G neuronal medium containing Neurobasal medium and 1× B27 supplement (Gibco), with 20 ng/mL GDNF (R&D Systems) and doxycycline. Doxycycline was removed on Day 10. Mouse embryonic stem cells (line ESD3) were differentiated to dopaminergic neurons by co-culturing with PA6 stromal cells according to a published protocol [26].

### Immunocytochemistry

Cells on glass coverslips were fixed in 4% paraformaldehyde with 0.15% picric acid, blocked in 10% chicken serum, 1% bovine serum albumin (w/v), 0.3% Triton X-100 in PBS and stained with antibodies to tyrosine hydroxylase (Millipore MAB318 or Santa Cruz sc-14007, both 1∶200), type III beta-tubulin (clone TUJ1, Covance MMS-435P, 1∶400), synaptophysin (Santa Cruz sc-9116, 1∶200), Girk2 (Santa Cruz sc-16135, 1∶50), Otx2 (Abcam ab21990, 1∶100), and V5 (Invitrogen R96025, 1∶400). Detection was performed with alexa-fluor labeled secondary antibodies (Molecular Probes) and coverslips were mounted in ProLong Gold Antifade Reagent with DAPI (Invitrogen). Imaging was performed on an Olympus iX-81 with Metamorph software. Quantification of TUJ1+ and Th+ cells was performed by counting a total of 3357 cells in 20 fields of view for astrocytes and 885 cells in 4 fields of view for fibroblasts.

### Magnetic sorting

Induced dopaminergic neurons containing the *MAP2*-CD4 reporter were grown on 10 cm dishes coated in poly-L-lysine. On Day 14, cells were harvested with Accutase (Sigma A6964), incubated with anti-CD4-conjugated MACSelect 4 Microbeads (Miltenyi Biotec 130-070-101) and sorted on MS columns (Miltenyi Biotec 130-042-201).

### RT-PCR

RNA was collected using the RNeasy Mini Kit (Qiagen 74104) and first-strand cDNA synthesis was performed using Superscript III reverse transcriptase (Invitrogen 18080051) with random hexamer primers. Quantitative real-time PCR was performed on an ABI 7900HT system (Applied Biosystems) using the TaqMan gene expression assays listed in Table 3 and normalized to beta actin (Mm00607939_s1). Analysis of gene expression data was performed with DataAssist version 3.0 (Applied Biosystems).

**Table 3:** TaqMan assays for RT-PCR.

Gene Symbol	Gene Product	TaqMan Assay
Aldh1a7	aldehyde dehydrogenase	Mm00496380_m1
Cacna1g	Cav3.1 calcium channel	Mm00486572_m1
Calb1	Calbindin-1	Mm00486645_m1
Ddc	DOPA decarboxylase	Mm00516688_m1
En1	Engrailed-1	Mm00438709_m1
Foxa2	forkhead box A2	Mm01976556_s1
Gabra1	GABA A receptor subunit alpha-1	Mm00439046_m1
Gad2	glutamic acid decarboxylase 2	Mm00484623_m1
Grin1	NMDA receptor 1	Mm00433800_m1
Kcnn3	KCa2.3 potassium channel	Mm00446516_m1
Lmx1a	LIM homeobox transcription factor 1 alpha	Mm00473947_m1
Mapt	microtubule-associated protein tau	Mm00521988_m1
Msx1	msh-like homeobox 1	Mm00440330_m1
Ngn2	neurogenin 2	Mm00437603_g1
Pax2	paired box gene 2	Mm01217939_m1
Pax5	paired box gene 5	Mm00435501_m1
Pitx3	paired-like homeodomain transcription factor 3	Mm01194166_g1
Pou3f2	POU domain, class 3, transcription factor 2 (Brn2)	Mm00843777_s1
Scn3a	Nav1.3 sodium channel, alpha subunit	Mm00658167_m1
Slc6a3	dopamine transporter	Mm00438388_m1
Slc17a7	vesicular glutamate transporter 1 (vGlut1)	Mm00812886_m1
Slc18a2	vesicular monoamine transporter 2 (Vmat2)	Mm00553058_m1
Syn2	synapsin II	Mm00449780_m1
Th	tyrosine hydroxylase	Mm00447557_m1

### Electrophysiology

Voltage- and current-clamp whole cell patch clamp recordings were performed on *MAP2*-GFP+ cells with neuronal morphology, visualized using an upright fixed stage microscope (Olympus) and a 40× water immersion objective. All recordings were performed at room temperature (20–24°C) using glass patch pipettes, fabricated on a Flaming-Brown micropipette puller (P-97, Sutter Instruments). The electrodes were 6–8 MΩ in resistance when filled with potassium gluconate-based intracellular solution (in mM: 145 K-gluconate, 2 MgCl_2_, 2.5 KCl, 2.5 NaCl, 0.5 GTP·Tris, 0.1 BAPTA, 2 ATP·Mg and 10 HEPES). The recording chamber was perfused with aCSF solution with the following composition: 155 mM NaCl, 3 mM KCl, 1 mM MgCl_2_, 3 mM CaCl_2_, 25 mM glucose and 10 mM HEPES, pH, 7.35. Neurons were recorded using an Axopatch 200B amplifier (Molecular Devices, Sunnyvale, CA), sampled at 10 kHz and digitized using Digitizer 1320A (Molecular Devices), with data stored for off-line analysis (Clampfit 10; Axon Instruments, Inc., Foster City, CA). In voltage clamp mode, cells were held at a membrane potential of −60 mV to record the potential pacemaker current fluctuations. To evoke voltage-gated currents, the command potential was held initially at −90 mV, then stepped to potentials ranging from −60 mV to +20 mV in 5 mV increments. In current clamp mode, cells were recorded for 2–10 min to examine spontaneous fluctuations in membrane potential and possible spontaneous firing. In order to calculate input resistance, all cells were injected with at least one hyperpolarized command current.

### HPLC for dopamine quantification

Membrane depolarization was evoked by incubating cells in a well of a 6-well dish with 1 mL aCSF containing 56 mM KCl for 15 minutes at 37°C. The aCSF solution was then collected and catecholamines were extracted onto 30 mg activated alumina (Wako 018-09561). The alumina was washed with ultrapure water and dried on Ultrafree-MC GV filters (Millipore UFC30GV00). Catecholamines were eluted into 100 µL 2% acetic acid (v/v) with 100 µM EDTA. 20 µL samples were analyzed on an HTEC-500 HPLC system with electrochemical detection (Eicom) using a reversed phase C18 separation column (Eicompak CA-5ODS). Mobile phase consisted of 88% 0.1 M phosphate buffer pH 6.0, 12% methanol, 600 mg/L sodium octanesulfonate, and 50 mg/L EDTA·2Na. Analysis was performed at 25°C with a flow rate of 230 µL/min and dopamine quantities were calculated by comparison of area under curve measurements to known standard dilutions of dopamine hydrochloride (Sigma H8502).

## Supporting Information

Figure S1 **Fibroblast-derived dopaminergic neurons.** (A) The ALN polycistronic vector is effective in converting fibroblasts to dopaminergic neurons, demonstrated by immunocytochemistry for the neuronal marker TUJ1 (14.9±2.3% positive) and the dopaminergic marker tyrosine hydroxylase (9.1±0.9% positive); the pan-nuclear marker DAPI is also shown. Scale bars 50 µm. (B) Action potential firing characteristics of fibroblast-derived dopaminergic neurons. Four overlapping traces are depicted derived from whole-cell current clamp recording of a representative induced dopaminergic neuron, elicited in response to hyperpolarizing and depolarizing current injection, increased from −20 to +10 pA in 10 pA increments. (C) Voltage-dependent sodium currents in fibroblast-derived dopaminergic neurons. Membrane potential was initially held at −90 mV and incrementally increased from −60 to +40 mV in 5 mV depolarizing steps. (D) Spontaneous action potential firing, consistent with a dopaminergic neuron pacemaker phenotype. The recording was conducted at resting membrane potential (−57 mV).
(TIF)

Movie S1 **Calcium oscillations in an astrocyte-derived dopaminergic neuron.** The genetically-encoded calcium indicator GCaMP3 is expressed under the control of the neuron-specific *MAP2* promoter. Fluorescence level increases with increased levels of intracellular calcium. Rhythmic oscillations of calcium levels are seen, consistent with the midbrain dopaminergic phenotype.
(WMV)
