# Supplementary figures and images for: Efficient Conversion of Astrocytes to Functional Midbrain Dopaminergic Neurons Using a Single Polycistronic Vector
Source: PLoS One. 2011 Dec 9;6(12):e28719. doi: 10.1371/journal.pone.0028719 (PMC3235158; doi:10.1371/journal.pone.0028719)

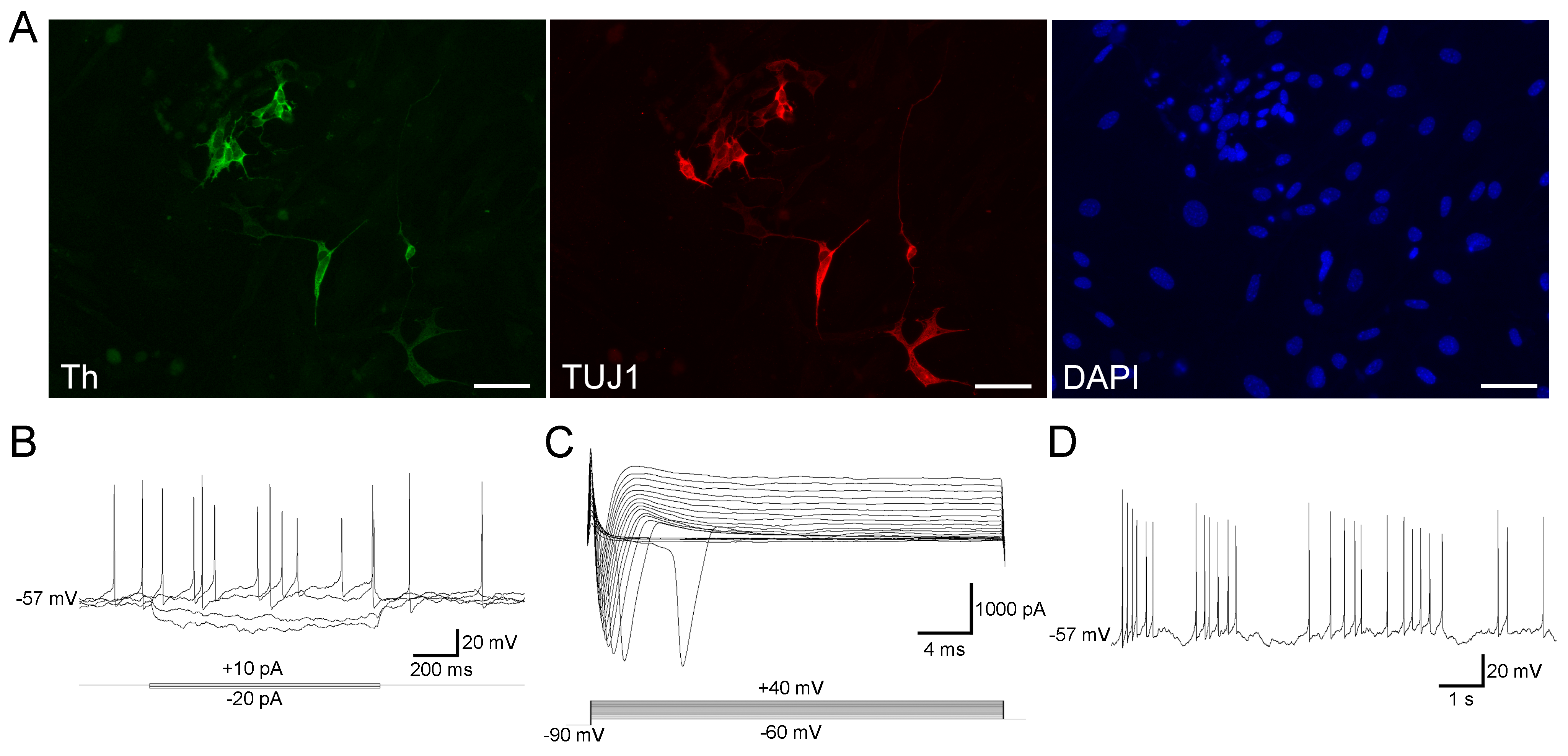

Supplement: Figure S1 — Fibroblast-derived dopaminergic neurons. (A) The ALN polycistronic vector is effective in converting fibroblasts to dopaminergic neurons, demonstrated by immunocytochemistry for the neuronal marker TUJ1 (14.9±2.3% positive) and the dopaminergic marker tyrosine hydroxylase (9.1±0.9% positive); the pan-nuclear marker DAPI is also shown. Scale bars 50 µm. (B) Action potential firing characteristics of fibroblast-derived dopaminergic neurons. Four overlapping traces are depicted derived from whole-cell current clamp recording of a representative induced dopaminergic neuron, elicited in response to hyperpolarizing and depolarizing current injection, increased from −20 to +10 pA in 10 pA increments. (C) Voltage-dependent sodium currents in fibroblast-derived dopaminergic neurons. Membrane potential was initially held at −90 mV and incrementally increased from −60 to +40 mV in 5 mV depolarizing steps. (D) Spontaneous action potential firing, consistent with a dopaminergic neuron pacemaker phenotype. The recording was conducted at resting membrane potential (−57 mV). (TIF) [file pone.0028719.s001.tif]
